# Interlaboratory Studies Using the NISTmAb to Advance Biopharmaceutical Structural Analytics

**DOI:** 10.3389/fmolb.2022.876780

**Published:** 2022-05-05

**Authors:** Katharina Yandrofski, Trina Mouchahoir, M. Lorna De Leoz, David Duewer, Jeffrey W. Hudgens, Kyle W. Anderson, Luke Arbogast, Frank Delaglio, Robert G. Brinson, John P. Marino, Karen Phinney, Michael Tarlov, John E. Schiel

**Affiliations:** ^1^ Institute for Bioscience and Biotechnology Research, National Institute of Standards and Technology, Rockville, MD, United States; ^2^ Agilent Technologies, Santa Clara, CA, United States; ^3^ National Institute of Standards and Technology, Gaithersburg, MD, United States

**Keywords:** monoclonal antibody, biopharmaceutical, nistmab, therapeutic protein, interlaboratory study

## Abstract

Biopharmaceuticals such as monoclonal antibodies are required to be rigorously characterized using a wide range of analytical methods. Various material properties must be characterized and well controlled to assure that clinically relevant features and critical quality attributes are maintained. A thorough understanding of analytical method performance metrics, particularly emerging methods designed to address measurement gaps, is required to assure methods are appropriate for their intended use in assuring drug safety, stability, and functional activity. To this end, a series of interlaboratory studies have been conducted using NISTmAb, a biopharmaceutical-representative and publicly available monoclonal antibody test material, to report on state-of-the-art method performance, harmonize best practices, and inform on potential gaps in the analytical measurement infrastructure. Reported here is a summary of the study designs, results, and future perspectives revealed from these interlaboratory studies which focused on primary structure, post-translational modifications, and higher order structure measurements currently employed during biopharmaceutical development.

## Introduction

Monoclonal antibodies (mAbs) have become the most prevalent biopharmaceutical modality, used to treat indications from viral infections to cancer. Numerous other protein-based drugs continue to evolve including antibody drug conjugates (ADCs), bispecifics, coagulation factors, and cytokines, among others. In addition, new modalities such as viral vector mediated gene therapies and vaccines, mRNA vaccines, and adoptive cell therapies have more recently emerged to fill previously unmet medical needs. Common to all modalities is the need for comprehensive structural characterization, identification of relevant critical quality attributes, and quality control of these features to maintain safety and efficacy. Lessons learned regarding analytical best practices for mAbs, perhaps the most widely characterized and understood from a structure-function perspective, can be ported to other modalities.

Comprehensive evaluation of the fundamental performance metrics and analytical capability of a technology are a pivotal first step prior to adapting, translating, or evolving measurement methods to new systems. Innovative analytical technologies are often performed to enable deep characterization, elucidate mechanisms of action, or better understand the intricacies of a manufacturing process. These emerging technologies, despite their potential for profound leaps in product or process understanding, may have limited historical precedence, thus presenting a barrier to rapid adoption. Interlaboratory studies may serve to lower these barriers by providing a means of harmonizing technical approaches, reporting community-wide performance metrics (i.e. precision), defining method best practices, and/or understanding the underpinning principles and sources of uncertainty in a measurement system.

Publicly available biopharmaceutical product-representative test materials are a pre-requisite to interlaboratory evaluation of community wide performance metrics. The NIST monoclonal antibody (NISTmAb) was introduced as a tool for advancing analytical methods pertaining to monoclonal antibodies. The NISTmAb reference material (RM) 8,671 is a recombinant humanized IgG1κ expressed in murine suspension cell culture that has undergone biopharmaceutical industry standard upstream and downstream purification to remove process related impurities. This RM is intended primarily for use in evaluating the performance of methods for determining physicochemical and biophysical attributes of monoclonal antibodies. It also provides a representative test molecule for development of novel technologies for therapeutic protein characterization. (Mouchahoir and Schiel, 2018; Schiel and Turner, 2018; Schiel et al., 2018; Turner and Schiel, 2018; Turner et al., 2018). The NISTmAb first debuted in a series of small interlaboratory characterization studies in 2015. (Schiel et al., 2014; Schiel et al., 2015a; Schiel et al., 2015b). This series of reports provided a useful baseline to identify measurements for which method advancement and regulatory assimilation would benefit from additional technology development and interlaboratory studies. Highly focused interlaboratory studies have since been reported by NIST and independent communities to harmonize best practices, deepen community consensus on method performance, and document a baseline performance upon which future method evolution may be based (DeLeoz et al., 2017; Hudgens et al., 2019a; Brinson et al., 2019; Coffman et al., 2020; Srzentić et al., 2020; Mouchahoir et al., 2021). A number of those interlaboratory studies are reviewed here, with the intention to spur future partnerships to target additional assays and/or method evolution through well-planned interlaboratory studies.

**FIGURE 1:**
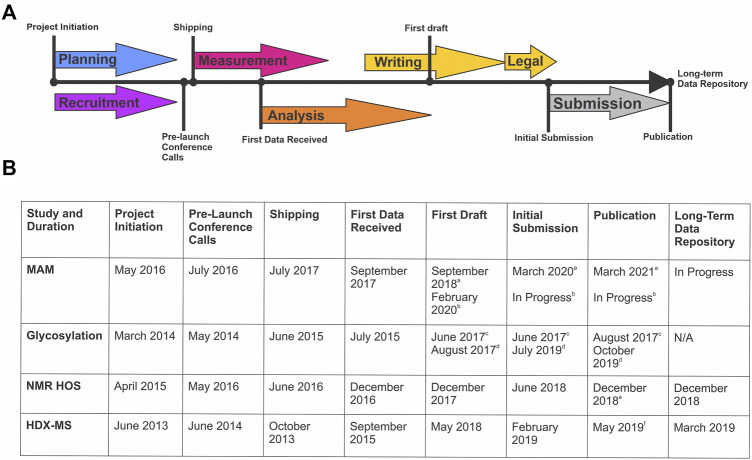
Timeline of Global NISTmAb Interlaboratory Studies. **(A)** Representative timeline identifying the key milestones for an interlaboratory study. **(B)** Corresponding dates and key milestones for each NISTmAb interlaboratory study (MAM, glycosylation, NMR HOS, and HDX-MS).^a^ MAM New Peak Detection Publication (Mouchahoir et al., 2021).^b^ MAM Attribute Analytics Publication (In progress).^c^ Glycosylation Interagency Internal Report (DeLeoz et al., 2017). ^d^ Glycosylation Interlaboratory Publication (De Leoz et al., 2020).^e^ NMR HOS Interlaboratory Publication (Brinson et al., 2019).^f^ HDX-MS Interlaboratory Publication (Hudgens et al., 2019a)

The design, coordination, implementation, writing, and publishing of each of these studies is an extensive community-wide effort that spans multiple years (Figure 1). Planning and recruitment stages of an interlaboratory study coincide and are often synergistic. Sample sets, measurement protocols, and reporting structures can evolve based on community feedback, considering study design is exceedingly difficult to change post-launch. Sample identity and preparation is a critical step most often performed by the study organizers, but in consultation with participants. This stage may involve alteration of material properties to “challenge” the analytical method and/or prepare the sample for analysis (i.e. digestion, mixing, or vialing). Samples must be suitable with respect to material properties for the intended use in the measurement system, be non-proprietary to enable public dissemination/publication of results, and be of sufficient stability, homogeneity, and purity.

The measurement phase of an interlaboratory study is conducted at each partners’ individual site. Participants are typically asked to complete a pre-defined report template and when possible, include raw data. Although reporting is templated, participation in such a study involves a significant commitment by participants and their parent organizations. The study design is intended to minimize financial and time commitment burden on the participants, but this aspect should not be overlooked as the intellectual engagement of experts in the field are critical to industry-relevant impact. Submitted datasets are most commonly anonymized via third party vendors to protect potential intellectual property, after which combined analysis of the anonymized data is conducted by the study organizers. Analysis, interpretation, and formulating the discussion around results are again a community effort involving all study participants. Numerous iterations of data analyses and participant feedback led to a first draft, initially approved by all co-authors, and then sent to partners legal for review. Use of a non-competitive material and data anonymization are critical to assure freedom to operate. Writing, submission, and acceptance can be a rather lengthy process to allow all authors and partner institutions to ultimately agree on the presentation and interpretation of results. Each of the studies reviewed herein are a consensus of 15–75 organizations. Interlaboratory studies represent broad industry commitment to achieve a high degree of unity and enable implementation of current best practices, evolve analytical methods, and expedite their uptake. A representative sampling of NISTmAb interlaboratory studies are reviewed here, specifically those that included one or more NIST organizers (multi-attribute method, glycosylation analysis, nuclear magnetic resonance, and hydrogen deuterium exchange interlaboratory studies). Each study had a slightly unique design and output, as necessitated by the intricacies of that particular method, which are reported herein to include the study Purpose and Method Description, Summary of Results, and Learnings and Future Perspectives. (Hudgens et al., 2019a; Brinson et al., 2019; De Leoz et al., 2020; Mouchahoir et al., 2021).

## Multi-Attribute Method Interlaboratory Study

### Purpose and Method Description

**FIGURE 2:**
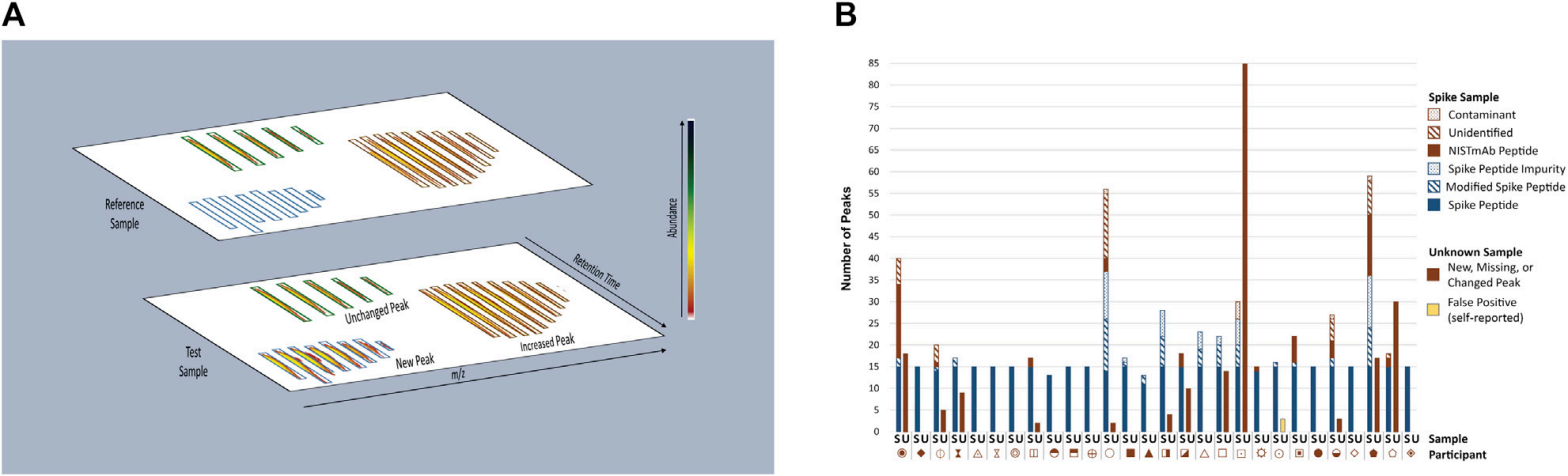
Overview of MAM New Peak Detection Data Analysis. **(A)** A representation of new peak detection data is shown for a single charge state/isotope cluster for a new peak, changed peak, and unchanged peak **(B)** Peaks Reported as New, Missing, or Changed in Spike and Unknown Samples. New, missing, and changed peaks detected in the Spike (S) and Unknown (U) Samples were reported by each participant. For the Spike Sample, peaks that conformed to expectation are represented in blue: Spike Peptides, Modified Spike Peptides (*e.g.,* Spike Peptide with a PTM) and Spike Peptide Impurities (*e.g.,* Spike Peptide with additional residue, truncation, etc.); peaks that did not conform to expectation are represented in red: NISTmAb Peptides, Unidentified Peaks, Contaminants. Peaks detected in the Unknown Sample did not conform to expectation and are represented in red without further categorization. One participant self-reported peaks in the Unknown Sample as false positives (represented in yellow) and thus were counted as a conforming result. Each participant is represented by a unique symbol. This figure was adapted from Mouchahoir et al., 2021 (https://pubs.acs.org/doi/10.1021/jasms.0c00415), with permission from ACS Publications; further permissions related to this material should be directed to ACS.

The multi-attribute method (MAM) builds upon industry experience with mass spectrometry (MS)-based peptide mapping (Formolo et al., 2015; Rogstad et al., 2017; Noor et al., 2020) and holds promise for use in the quality control (QC) space (Rogers et al., 2013; Rogers et al., 2015; Xu et al., 2017; Zhang and Guo, 2017; Rogstad et al., 2019; Millán-Martín et al., 2020; Sokolowska et al., 2020; Zhang et al., 2020; Mouchahoir et al., 2021). MAM is designed to monitor the status of pre-defined quality attributes within a therapeutic protein sample (*e.g*., post-translational modifications (PTMs), enzymatic clips, isomerization, *etc.*) and/or detect process impurities (*e.g.,* host cell proteins) in the sample. The basic workflow of a MAM platform begins with enzymatic digestion of the therapeutic protein, followed by separation of the resulting peptides by liquid chromatography (LC) and identification of the peptides by high-resolution mass spectrometry detection. Elegant software platforms are then used to interrogate the data to monitor changes in PTM levels within the sample (*i.e*., attribute analytics) and/or to detect impurities and unanticipated PTM changes in a non-targeted manner by comparison of the sample to a reference prepared in parallel (*i.e*., new peak detection (NPD). NPD is performed by first aligning reference and test sample data files according to *m/z* and retention time as depicted in Figure 2A. The data undergo “peak picking” where ions that meet a predetermined signal threshold (the new peak detection threshold) and display typical peptide isotope distributions are designated as peaks (bounded by blue, green or brown boxes in Figure 2A). The peaks detected in each sample are compared to the corresponding peaks (*i.e*., *m/z* and retention time match within a set tolerance) in the other sample. Peaks present in the test sample but not the reference sample are reported as new peaks, conversely peaks present in the reference sample but not in the test sample are missing peaks. If a peak is present in both samples and the difference in abundance between samples surpasses a set threshold (the fold-change threshold), it is reported as a changed peak. Unchanged peaks (below the fold-change threshold) are not reported. Prior knowledge of peak identity is not required; thus, NPD is an untargeted analysis that can potentially detect unexpected impurities or differences.

The potential for MAM to be implemented in the QC space as a replacement for a number of single-attribute assays has piqued the interest of the biopharmaceutical industry, and members of the industry are currently working to develop the platform for such use. Naturally, the adoption of new platforms comes with inherent risk which can slow the implementation of new technologies. The MAM interlaboratory study was therefore established to aid industry members at the beginning stages of MAM development and to provide a survey of the current performance of MAM across the industry (Mouchahoir et al., 2021). The study used the NISTmAb as a model therapeutic-like protein to evaluate the instrumentation and software processing used for MAM, and here we discuss the portion of the study that evaluated the NPD function of MAM. This was the first such industry-wide study to evaluate the performance of MAM.

**Study Design and Protocols**. Twenty-eight participating laboratories were recruited from members of the MAM Consortium (www.mamconsortium.org) and included representation from the industry, government, and software and instrument vendors. Each participating laboratory received a “kit” with the necessary materials for the study. The kit included four tryptic digests of the NISTmAb: one digest acted as the reference (Reference Sample) against which the other digests were compared for NPD, a second digest contained an additional set of 15 synthetic peptides spiked in to mimic impurities (Spike Sample), and a third digest was prepared from a NISTmAb sample that had first undergone high pH stress (pH Stress Sample) to test the untargeted analysis of changes in PTM levels. The fourth digest (Unknown) was the same sample as the Reference, however the identity was not revealed to the participants and served as a negative control. The kit also included a vial containing 15 synthetic peptides (Calibration Sample) to gauge instrument performance across laboratories and a vial of 0.1% formic acid in water for use as a blank injection to prepare the column.

Each participant followed the same LC-MS method and used the same column for sample analysis, but instruments and software packages for data analysis varied. The Calibration Sample was injected a total of three times, interspersed at the beginning, middle, and end of the queue. Participants were asked to report the retention time, observed mass, and summed extracted ion chromatogram (XIC) area for each Calibration Peptide. Each of the NISTmAb digests was injected twice. The first injection was acquired in MS-only mode to be used for the NPD analysis itself, and the second was acquired in MS-MS mode to be used for confident identification of peptides. Participants were asked to use their standard MAM analysis platforms on these samples and report any peaks detected as new, missing, or changed in abundance in the Spike, pH Stress, and Unknown Samples when compared to the Reference Sample.

### Results

**Instrument Performance: Calibration Sample.** ASTM Standard E691-18 (Standard Practice for Conducting an Interlaboratory Study to Determine the Precision of a Test Method) (ASTM, 2018) was used to evaluate the interlaboratory precision metrics of the retention times, mass accuracy, and fold-change values for each Calibration Peptide. Retention time repeatability standard deviations within each participating lab fell below 0.25 min, while reproducibility standard deviations between laboratories were measured between 1.4 min and 2.0 min. The larger variation in retention times between laboratories was expected due to the use of different LC systems. The high-resolution mass spectrometers used for the study achieved mass accuracy values of less than ±5 ppm, which is within the expected performance range for these instruments and is well within typical mass accuracy tolerance windows for database searching and NPD peak picking. Quantitative performance was measured by calculating the fold-change in abundance for each of the 15 peptides (*i.e*., ratio of a given peptide XIC from one injection to the XIC of the same peptide in another injection). Since the same volume of Calibration Sample was loaded onto the LC-MS system for each injection, the theoretical fold-change was 1 for each peptide. All but one of the absolute fold-change values for the Calibration Peptides averaged less than 1.26 with reproducibility standard deviations less than 0.35. Together, these performance metrics suggested that the instruments being used across the industry for MAM are performing within expected specifications.

**New Peak Detection: Spike and Unknown Samples.** Participants performed NPD on the Spike and Unknown Digests and reported any peaks that were new, missing, or changed in abundance (≥five-fold) when compared to the Reference Sample (Figure 2B). For the Spike Digests, fifteen of the participants conformed to expectation by detecting all 15 Spike Peptides as new peaks, with no additional new, missing or changed peaks reported (with the exception of synthetic impurities known to be inherent to the Spike Peptide mixture). Thirteen participants reported false positives (new, missing, or changed peaks that included NISTmAb peptides, unidentified peaks, and system contaminants), false negatives (fewer than fifteen Spike Peptides detected as new peaks), or both. Conformity to expectation for the Unknown Sample was met by sixteen participants who did not report any differences between the Unknown and References Samples. Peaks reported by non-conforming participants included NISTmAb peptides, unidentified peaks, keratin peptides (contaminants from the digestion process), a trypsin peptide, and one participant reporting carry-over of Spike Peptides.

The authors assigned a likely source for 92% of the non-conforming peaks for which a corresponding raw data file was available. This data evaluation showed the false positive peaks to be the result of 1) inadequacy of the column conditioning steps prescribed in the study protocol (causing large retention time shifts for a few NISTmAb peptides between the Reference and other samples and thereby interfering with peak alignment during software processing); 2) sample degradation (clipped peptides unique to four participants seemed to have been generated some time between shipment of the kit to the participants and injection onto the column); 3) system contamination (*e.g.,* plasticizer, trifluoroacetic acid adducts); 4) instrument-induced artifacts (*e.g*., in-source fragmentation, metal adduction); 5) peak abundance (low signal not well-distinguished from background); and 6) group-wise comparison of all four sample (rather than individual Reference to Sample comparisons; limited to one participant). False negative results (*i.e.,* Spike Peptides not reported as new peaks) were attributed to peak signal falling below the NPD threshold set during the peak picking process (*i.e*., distinguishing signal arising from true peptide peaks from noise), a value that was set according to each participant’s unique platform parameters.

**New Peak Detection: pH Stress Samples.** The degraded pH Stress Sample was expected to contain multiple new, missing, or changed peaks but the complexity of this sample did not lend itself to providing the authors with a definitive profile of expected differences to be found when compared to the Reference Sample. To survey the pH Stress Sample results the authors evaluated the consensus between peaks reported as new, missing, or changed by calculating the coincidence frequency (*ω* ^c^) (number of participants reporting a given peak) of each unique peak reported across laboratories and plotting the resulting values against the calculated peak coincidence population values [M(*ω* ^c^)] (number of peaks with the given coincidence frequency) (Hudgens et al., 2019a). The six highest *ω* ^c^ values ranged from 26 to 18 participants, each with a corresponding M(*ω* ^c^) value of 1 peak (Supplementary Figure S1). There were no peaks achieving the maximum possible *ω* ^c^ of 28 participants (*i.e*., no peak was reported by all participants). Processed NPD data files from one participant were available to aid our understanding of the low consensus values attained for the pH Stress Sample. These data showed incidences of new and changed peaks falling just below the participant’s NPD and fold-change thresholds, and co-elution with overlapping mass-to-charge ratios as the likely culprits.

### Learnings/Future Perspective

Evaluating the results of this interlaboratory study allowed the authors to gauge the performance of MAM across the industry and provide insights for improving the platform, especially for those in the beginning stages of developing their platforms.

The Calibration Sample provided a broad overview of instrument performance, with the results indicating that the instruments being used for MAM are performing within their expected ranges. Their performance, however, was not predictive of NPD performance in the other samples. The results of participants whose Spike and Unknown Sample analyses did not conform to expectation highlighted the importance of proper sample handling and instrument preparation (*i.e.,* column conditioning or washing) to ensure the integrity of the results. While in these cases the participants rightfully detected the differences between the samples as new peaks, these “nuisance positives” can cost valuable time and resources in a real-world situation as they would necessitate a follow-up investigation. The false positives generated by low abundant ions and the false negatives in the Spike Sample hint that NPD thresholds need to be carefully considered to strengthen the accuracy of the results. Investigation of the low consensus results for the pH Stress Sample also appear to have revealed FCD and NPD threshold settings as the primary source of differing results between participants. Although many participants used similar settings for their MAM evaluation they did not all achieve the same results, indicating that there is no universal threshold that may be applied across all instrument models, software platforms, or even samples.

Perhaps the key takeaway message from this study is that NPD can perform well in identifying new, changed, and missing peaks in the hands of many laboratories. MAM-specific system suitability protocols and criteria that include system performance, sample handling, and NPD/FCD thresholds may improve the success metrics. For example, a product-specific standard spiked with peptides representative of the product’s known process impurities and/or degradants could be run in parallel with the reference and product test samples. Appropriate system suitability sample design would include an empirical determination of appropriate process/product-specific impurity spike peptide quantities and associated NPD and FCD thresholds, considered in conjunction with desired process- and product-specific performance criteria. For example, the thresholds may be set by first finding the threshold value that is low enough to detect all spiked peptides, then continuing to lower that value as far as possible without generating any false positives. That NPD threshold could then be challenged by comparing process/product-specific reference and “unknown” samples and confirming that no new peaks are detected. A passing system suitability result for the optimized sample would require all spiked peptides to be detected as new or changed peaks with no additional peaks reported.

Overall, the MAM NPD interlaboratory study was a valuable way to understand the status of MAM throughout the industry, to identify potential pitfalls and provide guidance to users for improving their MAM methods. By taking the items discussed here into consideration the authors of the study believe that MAM NPD will be found ready for widespread implementation across the industry.

## Glycosylation Interlaboratory Study

### Purpose and Method Description

Glycosylation is the enzymatically driven covalent addition of monosaccharide residues to specific amino acids. These carbohydrates, known as glycans, play a crucial role in the safety and efficacy of therapeutic glycoproteins, including immunogenicity (Yehuda and Padler-Karavani, 2020), protein folding (Shental-Bechor and Levy, 2008), and thermal and protease stability (Zheng et al., 2014). Even with identical amino acid sequences, glycan alterations could arise during the manufacturing process of biologic drugs. Therefore, glycan characterization is critical.

Identification and quantitation of glycans is challenging due to their inherent heterogeneity in branching, linkage, and number of monosaccharide residues. To address this hurdle, laboratories employ a wide variety of derivatization, separation, identification, and quantification methods for glycosylation analysis.

**FIGURE 3:**
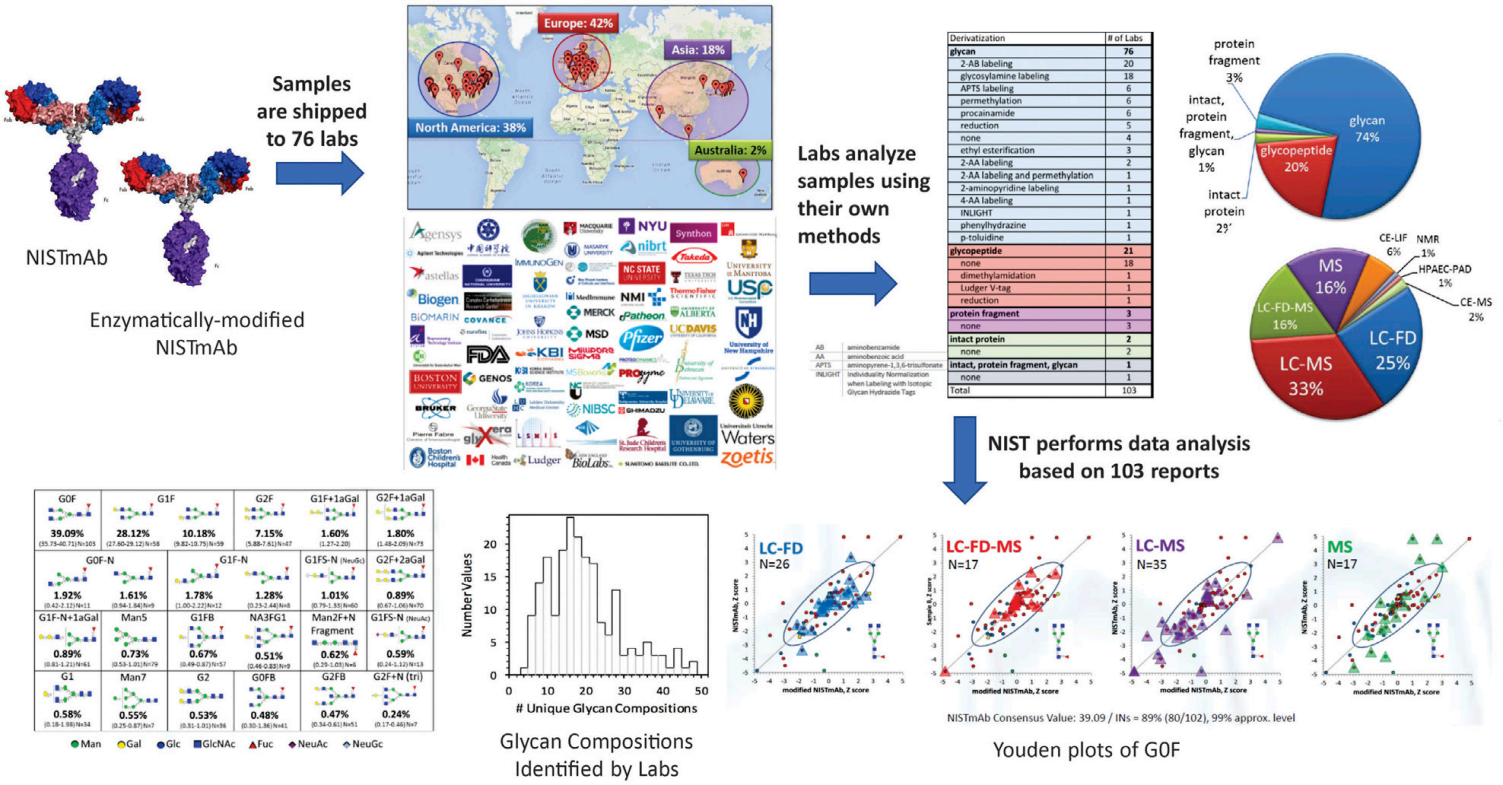
Schematic description of the glycosylation interlaboratory study. Participating laboratories used the glycoanalytical and detection method(s) of their choice to determine relative abundance of glycans.

**Study Design and Protocols**. The NIST glycosylation interlaboratory study (DeLeoz et al., 2017; De Leoz et al., 2020) was a broad-based interlaboratory study to determine the measurement variability of current glycosylation analysis methods. Participants included laboratories in biopharmaceutical companies, universities, research centers, government entities, and research hospitals, each of which chose their own measurement techniques. Laboratories analyzed enzymatically released N-glycans, digested glycopeptides, cleaved protein fragments or intact proteins to obtain glycan distributions (Figure 3).

NISTmAb has one site of N-glycosylation at the Fc region of the monoclonal antibody. Laboratories compared the glycosylation of two NISTmAb Primary Sample 8,670 samples: 1) NISTmAb and 2) 70% NISTmAb +30% NISTmAb without terminal β-1,4 gal glycans. Study participants were provided a spreadsheet to report the percent relative abundance of the glycans observed along with details associated with the method utilized for glycoanalysis. Data were analyzed without any normalization using several robust statistical analysis methods to measure variability and characterize glycan distributions. Having two similar samples enabled the formal separation of random from systematic variation using the two-sample Youden method (Youden, 1972).

### Results

**Overview of Methods.** Results were based on 103 reports from 76 laboratories in Europe, North America, Asia, and Australia. Laboratories employed a wide variety of techniques to characterize glycosylation: released glycans, glycopeptides, protein fragment or intact protein with or without derivatization. Detection methods included mass spectrometry, liquid chromatography, fluorescence detection, capillary electrophoresis, anion exchange chromatography, pulsed amperometric detection, nuclear magnetic resonance spectrometry, or a combination of these techniques.

Analysis of enzymatically released glycans is the most common technique. Industry laboratories most commonly used fluorescent labelling for released glycans but university laboratories preferred MS-based glycopeptide analysis or non-fluorescently labeled glycan analysis. On average, biopharmaceutical laboratories reported a lower number of glycan compositions than university laboratories. Differences in methods and number of glycans identified could be attributed to the laboratory’s objectives. For example, industry laboratories use validated chromatographic methods for a targeted set of glycans for QC and regulatory approval and limited their analyses to major glycans in mAbs. Alternatively, some groups in academia maximized the number of glycans they could identify for untargeted discovery.

Broadly, most laboratories used chromatography for separation followed by identification either by accurate mass or chromatographic retention times. Some laboratories combined both for identification. The number of glycan compositions reported by a single lab ranged from 4 to 48. Laboratories that employed MS only generally reported more glycan compositions. Laboratories that used MS with exoglycosidases, retention time (RT), fluorescence detection (FD) and/or MS/MS reported isomers. However, the range in the number of different reported compositions within each category was large.

**Glycan Identification and Quantification.** A total of 116 glycan compositions were reported by the laboratories, of which 57 compositions could be assigned community consensus abundance values. Supplementary Figures S2A,C summarize the percent abundances of the said 57 glycan compositions for A) NISTmAb and C) mod-NISTmAb. Only glycans reported at least six times for either NISTmAb or mod-NISTmAb are included in the plot. The glycan compositions are sorted in order of decreasing NISTmAb percent abundances.

Glycan compositions [h3n4f1], [h4n4f1] and [h5n4f1] are the most abundant compositions making up more than 85% of the total glycan abundance [see Supplementary Figure S2 caption for nomenclature for (glycan composition)]. Measurement repeatability is generally better for more abundant glycans. Horwitz observed that the interlaboratory CV is indirectly proportional to the analyte concentration no matter the analytical method or number of laboratories (Horwitz, 1982). This trend can be observed in Supplementary Figure S2A where the most abundant glycans on the left have tighter boxplots.

The dashed red line in Supplementary Figure S2B denotes the expected 1.0 ratio of mod-NISTmAb/NISTmAb when mod-NISTmAb and NISTmAb have similar glycan % abundances. Glycan compositions with terminal β-1,4 gal as their dominant structure are shown in red font in Supplementary Figure S2A,B,C. In theory, glycans with terminal β-1,4 gal (red font) should fall below this red line, *i.e*., they have lower percent abundance in mod-NISTmAb than in NISTmAb. As expected, Supplementary Figure S2B shows most of the reported glycans with terminal β-1,4 gal below the red line.

Each boxplot in Supplementary Figure S2A,B,C depicts the central 50% of the values with the horizontal middle line as the consensus median. The width of each box is proportional to the square root of the number of laboratories that identified that glycan. For example, 102 of the 103 data sets identified [h3n4f1] and [h4n4f1] and these two compositions have the widest boxes.

**Variability in Methods.** The boxplots of mod-NISTmAb/NISTmAb ratios in Supplementary Figure S2B display between-data set differences in the measurements of the two samples. Since the number and identity of the reported glycan compositions in the two samples were nearly the same within each data set, these ratios are not affected by normalization factors and could help demonstrate comparability.

Supplementary Figure S2D shows a target plot (Duewer et al., 1999) of the average variability and bias of the mod-NISTmAb/NISTmAb ratios in relation to the consensus medians. Each dot represents an aggregate score of a unique glycan composition in a result set. The *y*-axis shows the “concordance” or mean bias of the mod-NISTmAb/NISTmAb ratios calculated from $z_{i}=\left(\underset{j}{\sum }\left(x_{ij}-{\bar{x}}_{j}\right)/s_{j}\right)/n_{j}$ , where *x*_*ij*_ is the ratio of glycan composition *j* in the data set *i*, $\;{\bar{x}}_{j}$ is the consensus location of glycan composition *j*, *s*_*j*_ is the consensus dispersion for glycan composition *j*, and *n*_*j*_ is the number of data sets that report values for glycan composition *j* in both samples. Because the ratio distributions of most compositions are heavily-tailed, ${\bar{x}}_{j}$ is estimated using the median and *s*_*j*_ is estimated using the scale-adjusted median absolute deviation from the median (MAD_E_). the “apparent precision” of the biases or the bias estimate variability, estimated as SD, as shown in the *x*-axis:

$$s\left(z_{i}\right)=\sqrt{\underset{j}{\sum }{\left(\left(x_{ij}-{\bar{x}}_{j}\right)/s_{j}\right)}^{2}/\left(n_{j}-1\right).}$$

“Comparability” distances $\;d_{i}$ from (0,0), the ideal (*z*_*i*_, *s* (*z*_*i*_)) value, are depicted as semicircles:

$$d_{i}=\sqrt{z_{i}^{2}+s^{2}\left(z_{i}\right).}$$

The dots are colored based on their comparability distances: two comparability units are green, roughly indicating “Good” agreement with the consensus mod-NISTmAb/NISTmAb ratio estimates; between two and three units are yellow for “Moderate”; and greater than three units are red for “Questionable”. No systematic trend was observed when the target plot was examined by analyte, analytical technique, laboratory type, or number of replicates.

Youden two-sample analyses of glycan compositions were performed to distinguish random errors from systematic bias (Youden, 1972; Shirono et al., 2013). For example, most laboratories that used HILIC separation are within the univariate median for [h3n4f1] and [h4n4f1] glycans. For [h3n3f1], some laboratories that used HILIC are within the consensus median and some show the same proportional bias in both NISTmAb and mod-NISTmAb, which indicates a calibration issue. For [h5n4f1], laboratories that used HILIC show significant scatter, suggesting measurement challenges for this glycan composition.

**Community Consensus Medians**. The community consensus medians for NISTmAb glycosylation derived from this interlaboratory study (De Leoz et al., 2020) agree well with published NISTmAb glycosylation values (Formolo et al., 2015) for glycan abundance greater than or equal to 1%. Although it is challenging to get consensus at lower percent abundance, only three of the published values are outside the study’s central 50% distribution.

### Learnings/Future Perspective

This NIST glycosylation interlaboratory study provided a “snap shot” of the current state of measurement methods and precision for measurement of relative glycan abundances in a monoclonal antibody. Although the methods varied widely, agreement to the community consensus medians did not depend on a specific method, analyte, or laboratory type but on the measurement precision. Thus, ensuring within-laboratory repeatability is vital to the harmonization of glycosylation analysis methods between-laboratories. Methods used in the different laboratories could be corrected by calibration methods as appropriate standards become available. One such standard, NIST SRM 3655, was released in January of 2022 and is comprised of thirteen aqueous solutions of free reducing glycans commonly found in monoclonal antibody therapeutics. (SRM, 2021a). The certified mass fraction of each glycan was determined, thus enabling future studies to incorporate quantitative calibration and/or control materials.

The NIST glycosylation interlaboratory study provides a robust estimate of community consensus median relative values for NISTmAb glycosylation from an unmatched plethora of approaches applied to the same material. The values serve as a seminal starting point for comparing mAb glycosylation analysis methods. Further data mining studies on this large data set, such as comparing methods for identification, quantification or normalization, could help expose underlying systematic trends. Assigning degrees of confidence in identification with one, two, three, or four orthogonal values could be another area to explore.

The study warrants harmonization of glycosylation analysis methods. A thorough understanding of the sources of deviations could help harmonize methods for mAb glycosylation analyses. Less abundant glycan structures are challenging to identify especially without standards. In many cases, glycan structures are routinely assigned based on biological reasoning. The use of exoglycosidases helps narrow down potential hits but is of limited value for minor glycans or complex mixtures. The emerging field of ion mobility mass spectrometry could potentially aid in isomer separation and identification (Toraño et al., 2021).

## Nuclear Magnetic Resonance Higher Order Structure Interlaboratory Study

### Purpose and Method Description

The entirety of the structural elements from primary sequence to quaternary interactions has been termed the “higher order structure” (Almog et al., 1996) of a therapeutic protein. This critical quality attribute is essential for the safety and efficacy of these drugs, with deviation from the correct higher order structure (HOS) leading to lower product efficacy and adverse clinical outcomes (Fisher et al., 2016; Weiss et al., 2016). Currently, spectroscopic techniques (*e.g.*, Fourier transform infrared (FT-IR), differential scanning calorimetry (DSC), Raman, and circular dichroism (CD) that are predominately used to assess the HOS of a therapeutic cannot deliver high-resolution fingerprints of HOS and site-specific assignment of HOS perturbations. Development of robust, high-resolution analytical techniques that can be applied for HOS characterization throughout the lifecycle of a therapeutic protein, from development to manufacture, has therefore emerged as a major priority in the pharmaceutical industry.

To address this critical gap, two-dimensional nuclear magnetic resonance (2D NMR) methods have been developed to assess the HOS of a therapeutic at atomic level resolution (Aubin et al., 2008). While NMR methods were initially successfully implemented for small therapeutic proteins using ^1^H,^15^N heteronuclear correlations, it was unlikely that this NMR method could provide adequate sensitivity and resolution at natural isotopic abundance for mAbs (MW ∼ 150,000 Da) due to technical limitations arising from the high molecular weight, where slow molecular tumbling rate drastically reduces the resolution and sensitivity of the NMR measurement. Alternatively, the methyl group affords a faster internal rotational correlation due to the free rotation around the *sp* ^*3*^ bond axis, so that NMR signals from methyl groups can be observed effectively even for large proteins. Accordingly, ^1^H,^13^C NMR methyl fingerprinting methods were successfully implemented and determined to be sensitive reporters of overall protein fold, since methyl bearing amino acids are distributed throughout the protein molecule (Arbogast et al., 2015). Further studies indicated that even slight deviations in the glycoform distribution can be detected by this 2D NMR method (Arbogast et al., 2017).

Despite the successful implementation of methyl fingerprinting, wide adoption of this method for industrial HOS assessment required additional harmonization and demonstration of interlaboratory precision. Indeed, the 2D ^1^H,^13^C heteronuclear correlation experiment can be considered an NMR technique with many possible approaches for experimental implementation, including pulse sequence choice, acquisition parameters, and acquisition strategy (*e.g.*, uniform sampling versus non-uniform sampling). To fully harmonize the experimental methods and the analysis components of the 2D-NMR method, a global interlaboratory study was conducted with equal representation from academia, government, and industry, involving 26 laboratories from 9 countries, using the NIST-Fab, the Fab fragment derived from papain cleavage of the NISTmAb as the model therapeutic protein for the IgG1 molecular class (Brinson et al., 2019). In addition, two subsequent studies (Brinson et al., 2020; Sheen et al., 2020) followed, which used the interlaboratory data to further develop NMR processing and analysis tools well suited for biopharmaceutical applications.

**Study Design and Protocols**. The coordination, design, implementation, writing, and publishing of the NMR interlaboratory study was an extensive effort spanning nearly 4 years (Figure 1). The recruitment phase was launched officially at the CASSS-sponsored Higher Order Structure Meeting in April 2015 and took approximately 1 year. Equal representation was sought and achieved from industry, academia, and government laboratories. Further, 39 total magnets were represented in the study, ranging from 500 to 900 MHz, with at least two magnets represented at each field strength. In late May and early June 2016 four pre-launch conference calls were conducted to discuss the experimental protocols and receive feedback from partnering laboratories.

To ensure that the 2D NMR experimental protocol could be implemented at a field as low as 500 MHz, the NIST-Fab was used due to its smaller molecular weight compared to the intact molecule. A uniformly-labeled ^15^N, 20%-labeled ^13^C NIST-Fab was also produced from *Pichia pastoris* to serve as the system suitability sample (SSS). Each partnering laboratory was asked to perform one 2D ^1^H,^15^N gradient selected heteronuclear single quantum coherence spectroscopy (gHSQC) and six 2D ^1^H,^13^C gHSQC experiments using different acquisition strategies, including uniform sample (US) and non-uniform sampling (Magnusson and Ellison, 2008) and slightly different acquisition parameters. All partnering laboratories were also given the option to use different temperatures, pulse sequences such as the selective optimized flip angle short transient (SOFAST) heteronuclear multiple quantum coherence spectroscopy (HMQC), or their own laboratory experimental protocols. For data submission, all data packages were sent directly to a third party, National Association of Proficiency Testing (NAPT), who removed institutional identifiers from the submissions. This data anonymization was implemented to avoid bias for or against any partner.

**Public Web Data Repository.** The complete data package from the interlaboratory NMR study, included all blinded data, non-uniform sampling schedules, and processing scripts, was archived and is available at the following URL: https://www.ibbr.umd.edu/groups/nistmab-nmr

### Results

**Interlab Precision Analysis**. The precision of peak positions for both ^1^H,^15^N and ^1^H,^13^C gHSQC spectra was calculated using the combined chemical shift deviation (CCSD) and determined to be within the digital resolution of the measurement, 3.3 ± 1.8 ppb and 2.3 ± 0.8 ppb respectively, averaged across all magnetic fields. The ^1^H,^15^N CCSD value was consistent with the precision determined from an earlier interlaboratory NMR study on a small therapeutic protein (Ghasriani et al., 2016). Precision only degraded slightly from experiments performed with partner-generated NUS schedules and SOFAST-HMQC experiments; however, these experiments still had a very high precision of 5.3 ± 2.7 ppb. Indeed, these metrics point to the exquisite reproducibility and robustness of the NMR measurement, even for experiments for which deviations occurred from the established experimental protocol.

**FIGURE 4:**
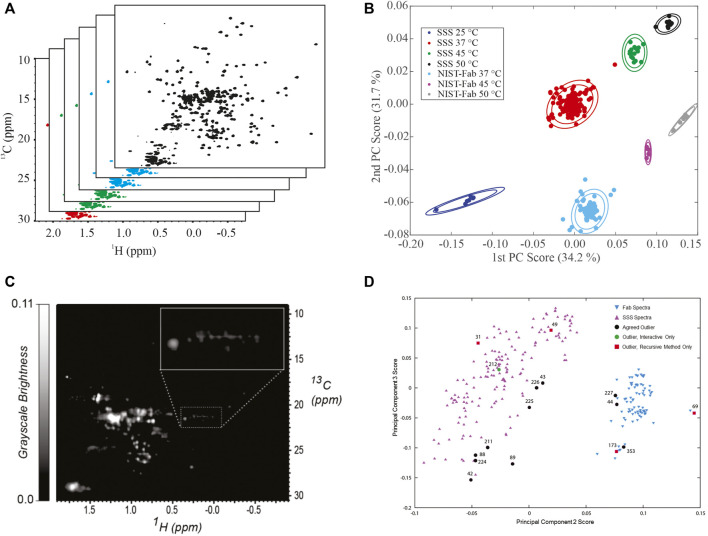
Representative Analysis of Data from NMR interlaboratory study. **(A)** simulated data package of many 1H, 13C methyl fingerprints; **(B)** PCA score plot of the interlaboratory NMR study; **(C)** Converted grayscale image of a 1H, 13C methyl fingerprint; **(D)** PCA score plot of 252 spectra used in the automated analysis of outliers. Panel 5B was reprinted from *Brinson et al.*, *2019* (https://doi.org/10.1080/19420862.2018.1544454), with permission from Taylor and Francis Group, LLC. Please note the article was published under a creative commons open access license. Permission is granted subject to the terms of the License under which the work was published. Panels 5C and 5D were Reprinted from Sheen et al., 2020 (https://doi.org/10.1016/j.chemolab.2020.103973) with permission from Elsevier.

**Principal Component Analysis.** Application of principal component analysis (PCA) to ^1^H,^13^C weighted peak tables afforded clustering of spectra into 7 groups by sample type, SSS versus NIST-Fab, and temperature (Figures 4A,B). All outliers could be explained by deviations from experimental protocol, such as temperature miscalibration, a custom NUS schedule, or another experimental set-up problem. As such, the PCA on peak position confirms the reproducibility of the measurement and shows the applicability of the method even at 500 MHz. Validation of a cluster assignment for a spectrum could be evaluated using the Dunn index or silhouette values (Brinson et al., 2020).

In addition to analyses of peak position, a 2D spectrum is a matrix of frequency-indexed points with intensity. In a follow-up manuscript to the original *mAbs* publication, the spectral matrixes of the methyl fingerprint region were used as input for PCA (Brinson et al., 2020). While seven clusters could still be identified, overlap of clusters was clearly observed. Further, a field dependence within PCA space was clearly identified, in part due to the intrinsic resolution differences between the multiple field strengths employed in the interlaboratory study. It is noted that the interlaboratory study was designed for harmonization of experimental protocols and the establishment of spectral similarity rather than addressing the issue of the field dependence in PCA analysis.

**Image Analysis and Automated Outlier Detection.** In addition to a matrix of frequency-indexed points, a 2D NMR spectrum was converted into a grayscale image followed by spectral classification with the Kullback-Leibler metric for spectral dissimilarity (Figure 4C) (Sheen et al., 2020). In this study using 252 ^1^H,^13^C gHSQC measurements from the interlaboratory study, the majority of spectra were properly classified using a recursive version of the automated method, which performed in a manner similar to human visual analysis (Figure 4D). In addition, this method detected three outliers that were initially missed by human eye but were determined to be true outliers after detailed analysis of each individual spectrum.

### Learnings/Future Perspective

The global NMR interlaboratory study benchmarked and harmonized the 2D NMR method to support its adoption for biopharmaceutical applications. While this specific case study focused on the NISTmAb, this method will also be amenable for small proteins, other mAbs, and other protein-based modalities. Indeed, the method was determined to be very robust: a highly similar answer is obtained despite slight deviations in acquisition protocol. These experimental variations include acquisition strategy (US vs NUS), pulse sequence choice, user, lab, or magnetic field. Further, this study has allowed for development of automated tools, including processing and spectral analysis. As an illustration of the acceptance of this method and utility of the benchmark data collected in the interlaboratory study, one NMR vendor has integrated the entire interlaboratory study package into its software for the basis of implementing chemometric analyses for biopharmaceutical applications. (Bruker, 2021).

When the interlaboratory study was designed, the NISTmAb was cleaved into its constituent domains, and the HOS of NIST-Fab evaluated to ensure the molecular size would be accessible at 500 MHz. Since this time, it has been established that the 2D NMR method is applicable to intact mAbs at fields as low as 600 MHz (Arbogast et al., 2017). Such a development is important, and it allows for minimal sample manipulation for many mAb-based therapeutics. Couple this with the Selective Excipient Reduction/Removal (SIERRA) filter to remove interferences from excipient signals (Arbogast et al., 2018), and the 2D NMR method allows for the assessment of this therapeutic class under many pharmaceutically relevant conditions.

Design of the NMR interlaboratory study primarily focused on experimental harmonization and creation of an NMR database for development of chemometric tools. While the initial analysis focused on peak positions, this approach requires visual analysis by an expert to define the peak lists, possibly resulting in biased evaluation. By contrast, analysis methods that use the total spectral matrix or images of the spectra can be performed automatically, although with potentially less specificity; PCA performed directly on the total spectral matrixes only showed loose separation into the expected clusters due to resolution difference from the multiple field strengths (Brinson et al., 2020). In the “real world” setting of the pharmaceutical laboratory, highly standardized HOS assessments will be performed on a single qualified magnet with validated protocols, which improves the performance of direct matrix analysis. Indeed, in two additional studies on intact NISTmAb, for which the field strength was controlled, PCA on the total spectral matrix of the methyl region detected very subtle changes resulting from alterations to the glycoform distribution as well as the concentration of formulation excipients (Arbogast et al., 2020; Brinson et al., 2020). Such an application overcomes the limitations of using the total spectral matrix of a chosen fingerprint region. The processing and analysis workflow could then be automated, making this information rich method accessible for the non-expert user.

The interlaboratory NMR study confirmed the repeatability and reproducibility of the measurement, and clearly established the technique as an effective tool to characterize HOS at all stages of therapeutic protein development and manufacturing. The data generated by the study has further helped to develop new analysis methods and to establish best practices. While this study provides a basis for defining spectral similarity, it remains an open question as to what degree of spectral perturbation is needed to affect a clinically meaningful change, and the degree of spectral response will likely be product specific.

## Hydrogen-Deuterium Exchange Mass Spectrometry Interlaboratory Study

### Purpose and Method Description

Hydrogen-deuterium exchange mass spectrometry (HDX-MS) is an established, powerful analytical tool for investigating protein-ligand interactions, protein folding, and protein dynamics (Engen et al., 2021). The great success of HDX-MS in these research areas has encouraged its development as a tool for QC of biopharmaceutical products.

Recent years have witnessed notable progress in the development of statistical methods for HDX-MS that will help realize QC applications; (Hourdel et al., 2016; Saltzberg et al., 2017; Weis et al., 2019; Hageman et al., 2021; Anderson et al., 2022); however, the effectiveness of statistical methods hinges on the quality of the HDX-MS measurements. For peptide level measurements HDX-MS measurement quality is characterized by protein sequence coverage and resolution, measurement variance, and absence of measurement bias.

The NIST interlaboratory HDX-MS project determined the reproducibility of continuous-labeling, bottom-up HDX-MS measurements (Gallagher and Hudgens, 2016; Hudgens et al., 2019a). Reproducibility is the precision of the analytical protocol after considering its application across different laboratories that have measured the same sample. Precision is just the closeness of agreement among measured values obtained by replicate measurements on the same or similar objects under specified conditions (JCGM, 2012). Precision is characterized by the components of intra-laboratory repeatability, intra-laboratory intermediate measurement precision, and inter-laboratory reproducibility (JCGM, 2012). Determinations of reproducibility allow for variations of instruments, reagents, locations, and operators.

An understanding of reproducibility is necessary for the use of HDX-MS in commerce, such as QC of biopharmaceutical production, lot qualification, and acceptance of a determination of similarity between a biosimilar candidate and its innovator therapeutic protein. Quality control of a biopharmaceutical requires that measurement criteria of its critical quality attributes (CQA) remain stable over the 20 + year lifetime of a biopharmaceutical product. During this period QC laboratory location, personnel, and instrumentation will undoubtably change.

To gain insight into the degree of variation that a biopharmaceutical QC laboratory will encounter, NIST initiated the interlaboratory HDX-MS project which recruited 15 laboratories to contribute HDX-MS measurements on a standardized sample of NIST-Fab. Information gained from a reproducibility determination can guide the selection of operation protocols and apparatus during establishment of an HDX-MS quality control laboratory.

**FIGURE 5:**
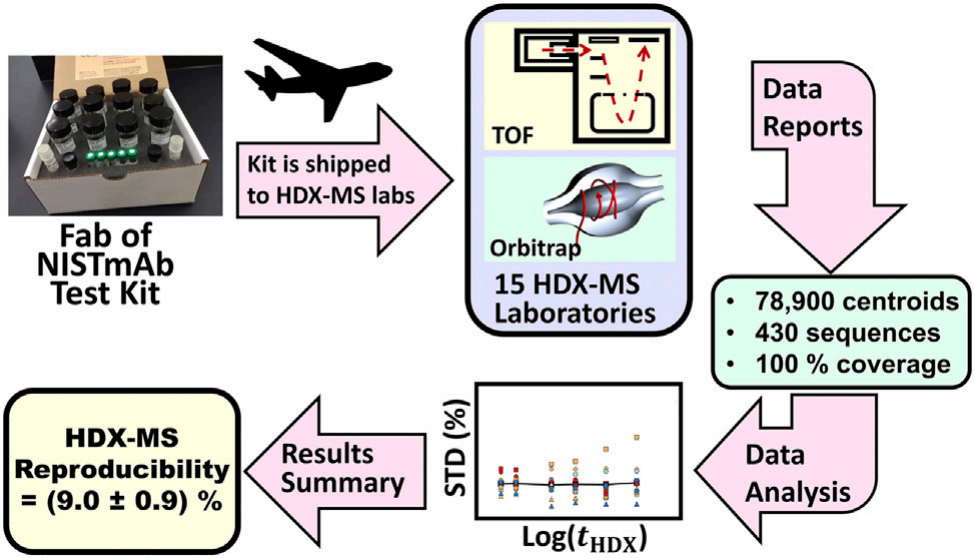
Schematic description of the HDX-MS interlaboratory study. Fab of NISTmAb test kits containing standardized solutions were overnight shipped to participating laboratories. Laboratories measured peptic peptide centroids and reported these results to NIST. NIST evaluated the accumulated data to determine interlaboratory precision.

**Study Design and Protocols.** During the NIST interlaboratory HDX-MS study, each laboratory received a standardized kit that contained buffered solution of Fab fragment of NISTmAb reference material (PDB: 5K8A), (Marino et al., 2015; Karageorgos et al., 2017; Gallagher et al., 2018), and vials of buffers and reagents used during the sample labeling, sample denaturing, and quenching steps (Figure 5). The kit harmonized solution pH, salt concentration and disulfide bond reducing power.

During the HDX-MS interlaboratory project the laboratories and investigators were permitted use of any instrumentation and software. The laboratories were not directed to report specific peptide sequences nor were they told the deuterium uptake rates of previously observed peptides. As required by the instrumentation incumbent in each laboratory, operators adjusted protein and deuterium concentrations, and each operator selected one of four D2O bath temperatures; $T_{\text{HDX}}\left(Lab\#\right)$ = 25°C, 24°C, 21°C, and 3.6°C; resulting in a diverse powerset spanning a range of operating conditions.

**Public Web Data Repository.** A complete package of anonymized data from the interlaboratory HDX-MS study, is publicly available. (Hudgens et al., 2019b).

### Results

**Protein sequence coverage.** Each laboratory conducted proteomics studies on the NIST-Fab sample and reported centroid measurements of peptides manifesting strong intensity and an absence of interference from co-eluting peaks. The laboratory cohort used pepsin protease to digest Fab of NISTmAb into 609 peptide ions originating from 430 sequences of the light and heavy chains. On average, laboratories reported 103 peptide sequences; however, datasets ranged between 41 and 175 peptide sequences, giving ≈60–99% sequence coverage of the Fab protein. Despite this dispersion in the number of peptide sequences reported by laboratories, an analysis determined that the six instrument-software configurations share nearly equal capacity to detect and identify peptides from NIST-Fab.

The commonality of like sequences across all datasets was characterized by coincidence frequency $\omega^{c}$ , the number of laboratories reporting a specific peptide sequence, and $M\left(\omega^{c}\right)$ , the sequence coincidence population, which is the number of peptide sequences of each coincidence frequency, $\;\omega^{c}$ . For example, M(1) = 245 is the population of sequences found only once among all laboratory datasets. Across the laboratory cohort, $M\left(\omega^{c}\right)$ falls rapidly with increasing coincidence frequency, such that only two peptide sequences are reported by all laboratories, *i.e*., M(15) = 2 (Figure S3). In fact, nearly 50% of all reported sequences are unique, *i.e*., $\omega^{c}$ = 1.

**Precision.** Each laboratory submitted spreadsheets listing centroids, ⟨*m* (*t*_HDX_)⟩, for each peptide at *t*_HDX_ = 0, 30, 60, 300, 900, 3,600 s, 14,400 s. The spreadsheet also listed reference centroids for each peptide, ⟨*m* (*t*_HDX_ = ∞_pseudo_)⟩, from a perdeuterated control sample. The 15 data sets contain ≈78,900 centroid measurements of the heavy and light chains of the Fab fragment, which differences, ⟨*m* (*t*_HDX_)⟩—⟨*m* (*t*_HDX_ = 0)⟩, yield *D* (*t*_HDX_)’s. The record for each peptide comprised three “runs”, each conducted on a different day. Each “run” comprised three replicant centroid measurements, termed “reps”, conducted on the same day. Plots of variances of *D* (*t*_HDX_) for all peptides showed that most laboratories (87%) achieved centroid mass laboratory repeatability precisions of ⟨*s* ^Lab^⟩ less than or equal to (0.15 ± 0.01) Da (1σ_x̅_), where σ_x̅_ is the standard error of the mean. All laboratories achieved ⟨*s* ^Lab^⟩ less than or equal to 0.4 Da. Plots of *D* (*t*_HDX_) variance vs. *t*_HDX_ demonstrated that such plots can detect problems with instrumentation and procedures.

To account for the diverse solution environments of the different laboratory settings, analyses of reproducibility used $\text{\%}E_{\text{corrected}}^{\text{peptide}}\left(t_{\text{HDX}}\right)$ , which includes an adjustment for H for D back-exchange during the quench and analysis procedures and a correction for non-unitary deuterium fractions in the exchange solution, $F^{{\text{D}}_{2}\text{O}}$ (≡ $\%D_{2}O/100\;\%$ ): (Zhang and Smith, 1993; Mayne, 2016):

$$\text{\%}E_{\text{corrected}}^{\text{peptide}}\left(t_{\text{HDX}}\right)=\;\frac{D^{\text{peptide}}\left(t_{\text{HDX}}\right)\cdot 100\text{ \%}}{F^{{\text{D}}_{2}\text{O}}\left({\langle m\left(\infty \right)\rangle }^{\text{peptide}}-{\langle m\left(0\right)\rangle }^{\text{peptide}}\right)}$$

where $m{\left(\infty \right)}^{\text{peptide}}$ , approximated by $m{\left(\infty_{\text{pseudo}}\right)}^{\text{peptide}}$ , is the centroid mass of a peptide from a protein sample containing only deuterons at its amide sites.

For immersions of protein at *T*_HDX_ = (3.6–25) ^o^C and for D_2_O exchange times of *t*_HDX_ = (30 s–4 h) the reproducibility of back-exchange corrected, deuterium uptake measurements for the 15 laboratories is $\sigma_{\text{reproducibility}}^{15\text{ Labs}}\left(t_{\text{HDX}}\right)$ = (9.0 ± 0.9) % (1σ). A nine-laboratory cohort that immersed samples at *T*_HDX_ = 25 ^o^C exhibited reproducibility of $\sigma_{\text{reproducibility}}^{25\text{C cohort}}\left(t_{\text{HDX}}\right)$ = (6.5 ± 0.6) % for back-exchange corrected, deuterium uptake measurements.

**Factors affecting HDX-MS measurement precision.** Main effects analyses of *mean response* (mean deuterium uptake) of peptides as a function of solution and operational variables can suggest contributions leading to increased measurement variance (Filliben et al., 2003). For several variables *mean response* changed in accord with theory and design of the experiment. As examples, peptide exchange *mean response* increased from 18 to 48% between *t*_HDX_ = 30 s and *t*_HDX_ = ∞_pseudo_, and *mean response* of peptides were distinct, as expected for sequences residing in different local structural environments. A procedure used by some laboratories of flash freezing protein samples immediately after the *t*_HDX_ period expires was shown to have no adverse effect on the *mean response*. *Mean response* for the variable, “*run*#” was essentially constant, indicating that laboratory platforms maintained stable, day-to-day solution and temperature environments.

Main effects analyses revealed non-ideal *mean responses* for variables, $T_{\text{HDX}}$ , *Lab#*, and *%D*_*2*_ *O.* Since amides undergo ≈3 × increases in exchange rates for each increment of 10 ^o^C, (Jensen and Rand, 2016), $\text{\%}E_{\text{corrected}}^{\text{peptide}}\left(t_{\text{HDX}}\right)$ should increase smoothly with increasing $T_{\text{HDX}}$ . Contrarily, a jagged response pattern is observed for laboratories reporting $T_{\text{HDX}}$ = 22 ^o^C, 24 and 25^o^C, suggesting that the reported exchange bath temperatures may differ from the true temperatures. Although stable temperature can be maintained by ice baths or electronic temperature stabilizing equipment, verification that $T_{\text{HDX}}$ reported by sensors is the true temperature requires regular calibration against a temperature standard traceable to a recognized standards organization (Nicholas and White, 2001; SMR, 2021b). The HDX-MS kit instructions did not require verifications of the true $T_{\text{HDX}}$ . Hence, laboratories reporting the same $T_{\text{HDX}}$ may have acquired data at several temperatures, leading to dispersions of *D* (*t*_HDX_). Similarly, the *mean response* was expected to be invariant with *%D*_*2*_ *O*; however, the sparse sample size for each dilution value correlated *%D*_*2*_ *O* tightly with *Lab#*. Regardless, the variance of *mean response* for *%D*_*2*_ *O* result suggests that volumetric dilution accuracy may vary among laboratories.

*Mean response* for the variable, “*rep*#” exhibited ≈3.5% reduction between the first and third “*rep*”. Since each series of “*reps*” is executed within the same run, the steady reduction of response is evidence for increasing accumulation on columns of residual peptides from previous chromatographic analyses that have completely back-exchanged (Fang et al., 2011; Majumdar et al., 2012).

### Learnings/Future Perspective

The HDX-MS interlaboratory comparison provided guidance that can be used to improve the acquisition of HDX-MS data. For short-term projects, such as mapping of protein-ligand interactions, the main effects analyses suggest a need for more precise control over the volumetric fractions that determine the exchange bath deuterium content and aggressive washing of the protease, trap, and analytical columns between chromatography runs. The study also showed that repeatability plots of average measurement deviation vs. $t_{\text{HDX}}$ can detect procedural problems during measurement campaigns. For protein folding experiments knowledge of true $T_{\text{HDX}}$ will improve derivations of thermodynamic properties.

Information gained from the NIST interlaboratory determination of reproducibility can guide the selection of operation protocols and apparatus during establishment of an HDX-MS quality control laboratory. The reproducibility, $\sigma_{\text{reproducibility}}^{25\text{C cohort}}\left(t_{\text{HDX}}\right)$ = (6.5 ± 0.6) %, is likely sufficient for many QC programs, and the observation that contemporaneous instrument-software combinations can achieve this precision should embolden the use of HDX-MS for QC applications. Recommendations for hardware and protocol improvements given above will further reduce the uncertainty budget of HDX-MS.

The observation that sequence coincidence population $M\left(\omega^{c}\right)$ falls off rapidly with the number of reporting laboratories, where $\omega^{c}$ is the number of measurement instances during a product lifetime, indicates that the QC laboratory is unlikely to be successful if it relies only on a chromatography system to elute the same set of peptides over years. The potential for failure exists because datasets containing ≈ 250 peptide sequences are much smaller than the 8,100 peptide sequences containing 4 to 30 amino acids predicted by an *in silico* digestion calculation of NIST-Fab. Over the short-term, instrument and operator bias will favor observation of the same peptide sequences, but as HDX-MS system conditions change slightly, the somewhat stochastic behavior of pepsin will cause new peptides to appear and others to disappear. Although the total number of peptide sequences in each dataset may remain unchanged, not all sequences may be available for comparison with the reference peptide sequence set.

Prospective QC laboratories can select instrumentation and protein models that can sidestep the obstacle presented by comparing datasets containing few peptide sequences that are the same as observed for the HDX-MS dataset, derived for the biopharmaceutical reference material. First, mass spectrometry instrumentation provisioned with electron transfer dissociation (ETD) (Landgraf et al., 2012; Huang and Hudgens, 2013; Jensen et al., 2015; Hamuro, 2017) and/or ultraviolet photodissociation (UVPD) (Mistarz et al., 2018) can trim larger peptides into smaller peptides and allow subtraction of the deuterium content from post translational modifications. Thus, the QC laboratory can modify the mass spectrometer data acquisition procedures to subject eluting peaks to ETD and UVPD and produce an ensemble of sequences that match the reference material dataset. Secondly, the QC laboratory can construct a dynamics model of the therapeutic protein by observing many overlapping peptides, such that single-amide resolution D-uptake rates are known of the entire sequence. This dynamics model could accommodate comparisons involving nearly any ensemble of peptide sequences.

To facilitate HDX-MS measurements of improved accuracy, precision, and greater peptide coverage, NIST has developed a HDX-MS chromatography apparatus that addresses metrology problems found during the HDX-MS interlaboratory comparison (Hudgens, 2020). To expand the size and sequence coverage in HDX-MS datasets, this apparatus can automatically switch between two distinct protease columns to produce two distinct peptide sequence ensembles during a single run. To eliminate noise and chromatographic carryover from aggregates and agglomerates, quaternary pumps flush and backflush protease, trap and analytical columns with various cleaning solutions. While the chromatographic gradient elutes peptides from the trap and separates them on the analytical column, the protease column undergoes an additional backflush cleaning cycle. Idle columns are stored in place and are perpetually cleaned and conditioned. To minimize back-exchange and also maximize the number of sequences in datasets, protein proteolysis is conducted at (0 ± 0.06)^o^C, and the trap and analytical columns separate proteolytic peptides at (-30 ± 0.02)^o^C, permitting chromatography runs as long as 0.75 h. The expanded analysis gradient better separates chromatographic peaks, resulting in lower peak overlap and larger dataset coverage.

Temperatures within HDX-MS apparatus can become poorly regulated, due to elevated temperature in the site facility. However, the large thermal mass and liquid coolant system of this HDX-MS instrument maintains internal zone temperatures at their setpoints as the laboratory temperature varies from 20°C to 30°C. These improvements to HDX-MS instrumentation, stimulated by findings of the HDX-MS interlaboratory comparison project, will improve HDX-MS metrology, in general, and facilitate the development of QC facilities in the biopharmaceutical industry.

## Global Discussion/Future Perspectives

Interlaboratory testing to evaluate similarity of results achieved by multiple laboratories performing the same analytical measurement is a fundamental concept in international metrology. A variety of documentary standards such as ISO 17025, ISO/TS 21748 and ASTM D7778-15 are available to assist in guiding design and implementation of an interlaboratory study to obtain consensus values, precision estimates, and establish if a given laboratory in a cohort has a systematic bias or is in control with community performance. Key comparisons are invaluable in interlaboratory studies, particularly in international metrology as part of the International Bureau of Weights and Measures (BIPM). As part of their mission to promote global comparability of measurements, the BIPM coordinates, in consultation with internationally representative committees, a variety of international key measurement comparisons to facilitate international trade and scientific discovery. Historically, these key comparisons involve measurements that are traceable to a fundamental unit of measurement (*i.e*., kg, second). The Protein Analysis Working Group (PAWG) for example, coordinates studies on the ability to perform absolute concentration determination of biomolecules in complex matrices. (Josephs et al., 2019). More recently, this working group has begun to discuss physicochemical measurement properties as possible additions to key comparisons as a response to the increasingly vital nature of such measurements to industrial biotechnology (CCQM, 2021).

The NISTmAb interlaboratory studies reviewed herein include participation from a broad sampling of biopharmaceutical companies, federal stakeholders, instrumentation vendors, and academic experts. The intent of the studies has slightly orthogonal drivers in that metrological traceability and/or analytical proficiency is not the main driver; however, their impact on fostering international agreement, and thus industrial impact, should not be overlooked. The availability of the NISTmAb has spurred this series of rather unique interlaboratory studies. The NISTmAb and other biopharmaceutical products are inherently complex materials for which measurements are continuously evolving. Interlaboratory studies conducted on NISTmAb to date fall into a few broad categories based on their intended purpose: 1) Survey of analytical approaches, 2) Technical performance evaluation, 3) Harmonization and/or analytical proficiency to enable platform adoption. Of those studies conducted, the current interlaboratory studies described herein fit into the first and second categories.

Interlaboratory studies for surveying analytical approaches are designed to better understand the nature and variety of analytical approaches being applied by a community for a given measurand. For example, a battery of methods has been developed for measurement of glycans, and was a driver in the inception of the NISTmAb platform (Schiel et al., 2012). A few of these analytical methods were applied to NISTmAb in a 3-lab study as part of its initial characterization (Prien et al., 2015). The glycosylation interlaboratory study expanded the scope to a comprehensive, industry-wide example of a survey-based interlaboratory study. Study participants used a variety of sample preparation and analysis approaches ranging from intact antibody, fragments, glycopeptides, or released glycans. The full range of available sample preparation, data acquisition, and results interpretation strategies were reported. The global interlaboratory study informed the broader community of what methods were being employed where, such that a more informed decision may be made to determine which methods are most suitable for a given laboratory’s intended use (De Leoz et al., 2020).

The remaining studies reviewed herein have been geared toward technical evaluation of a given analytical method. In a typical method validation performed to meet ICH Q2 (R1) (Guideline, 2005), the between-site reproducibility is required to set appropriate specifications and it is typically performed harmonizing all equipment, operating procedures, samples, etc. The MAM, NMR, and HDX-MS studies sought to harmonize a significant number of steps that may include sample preparation, instrument settings, and/or data analysis, while other aspects such as analyst, equipment, and software were at the participants’ discretion A goal of these experiments was to reduce the number of variables and hone in on specific aspects of the particular measurement system that may contribute to measurement uncertainty. Each study had a slightly unique design and output, as necessitated by the intricacies of that particular method, yet every study enabled reporting of community-wide performance metrics (i.e. precision, robustness, detection limits, etc.). It should be noted that these community performance metrics do not represent product specifications that can be utilized in a pass/fail mode, but rather they present preliminary milestones in analytical performance when using the NISTmAb as an external system suitability control and/or developing a method for an in-house proprietary material. More importantly, the common sources of uncertainty along with potential mitigation strategies were identified. Such information is valuable to assure proper procedural controls and identify opportunities for continuous method improvement.

Provided sufficient evolution and acceptance, each of these methods could 1 day become routine measurements performed on mAbs and other protein systems. In this case, the third and final category of interlaboratory harmonization study may be performed. Platform adoption would be enabled by broadly expanding measurements to numerous analytes, users, etc., wherein participants would follow all previously determined best practices. A higher order measurement system (i.e., primary calibrators, system suitability controls, etc.) would enable determination of values and associated uncertainty estimates representative of true interlaboratory variation. Interestingly, some measurements we are describe with these multifaceted approaches, such as structure and structure-function relationships, may not resolve to a single well-defined number and associated uncertainty. However, the antithesis of the preceding statement, is that novel data reduction strategies may in fact 1 day provide a single similarity scoring value, a feat in part made possible by availability of evolved analytical measurement best practices. (Arbogast et al., 2020; Brinson et al., 2020).

While the intended purpose of an interlaboratory study supporting protein structure measurements may differ, the need for a representative test material remains constant. Such a material must be well characterized, stable, and homogeneous with respect to the relevant material attributes must be utilized. Each measurand may not necessarily be well characterized and available on the certificate, in fact for technology development interlaboratory studies the intended measurand is likely not part of an assignable measurement system as are traditional metrological values. In this circular reality of measurement science, broadly available test materials support measurement innovation, while measurement innovation increases product and manufacturing process understanding. Through numerous publications, including the interlaboratory studies described herein, the NISTmAb RM 8671 has been shown to be a valuable test material for interlaboratory studies. Its properties along with use cases highlight some of these important characteristics. The test material to be utilized should also be available long term with an appropriate quality system to ensure stability and continuity of material properties. This is typically achieved using cold storage and a continuous testing strategy. The ideal material would be a Reference Material developed following ISO Guidelines. If batches or lots are necessary, as they may well be for future materials available in only small quantities, an uncertainty evaluation considering the potential for inter-lot variation and drift should be considered and accounted for by the sponsoring institution. By no means is this an exhaustive list of considerations for an appropriate interlaboratory material and associated study design, but the intent is to highlight major considerations before delving in to the specific measurement system to be evaluated.

## Conclusion

The availability of the NISTmAb has enabled a series of interlaboratory studies that have a dual-intended purpose: 1) to allow open information sharing to discuss experiences among companies, government agencies, and academicians alike, 2) to assist scientists in making informed decisions when selecting an analytical method, as well as appropriate sample preparation, data acquisition, and data analysis settings. It is likely that interlaboratory studies like those employing NISTmAb can be similarly used to assess the state-of-the-art of analytical characterization of new and emerging modalities. Additional pre-competitive materials representative of a given product class, as well as targeted interlaboratory studies to evaluate their properties and measurement bias, will be critical as the landscape of both pharmaceutical modalities and analytical methods continues to evolve.
