# Supplementary figures and images for: Interlaboratory Studies Using the NISTmAb to Advance Biopharmaceutical Structural Analytics
Source: Front Mol Biosci. 2022 May 5;9:876780. doi: 10.3389/fmolb.2022.876780 (PMC9117750; doi:10.3389/fmolb.2022.876780)

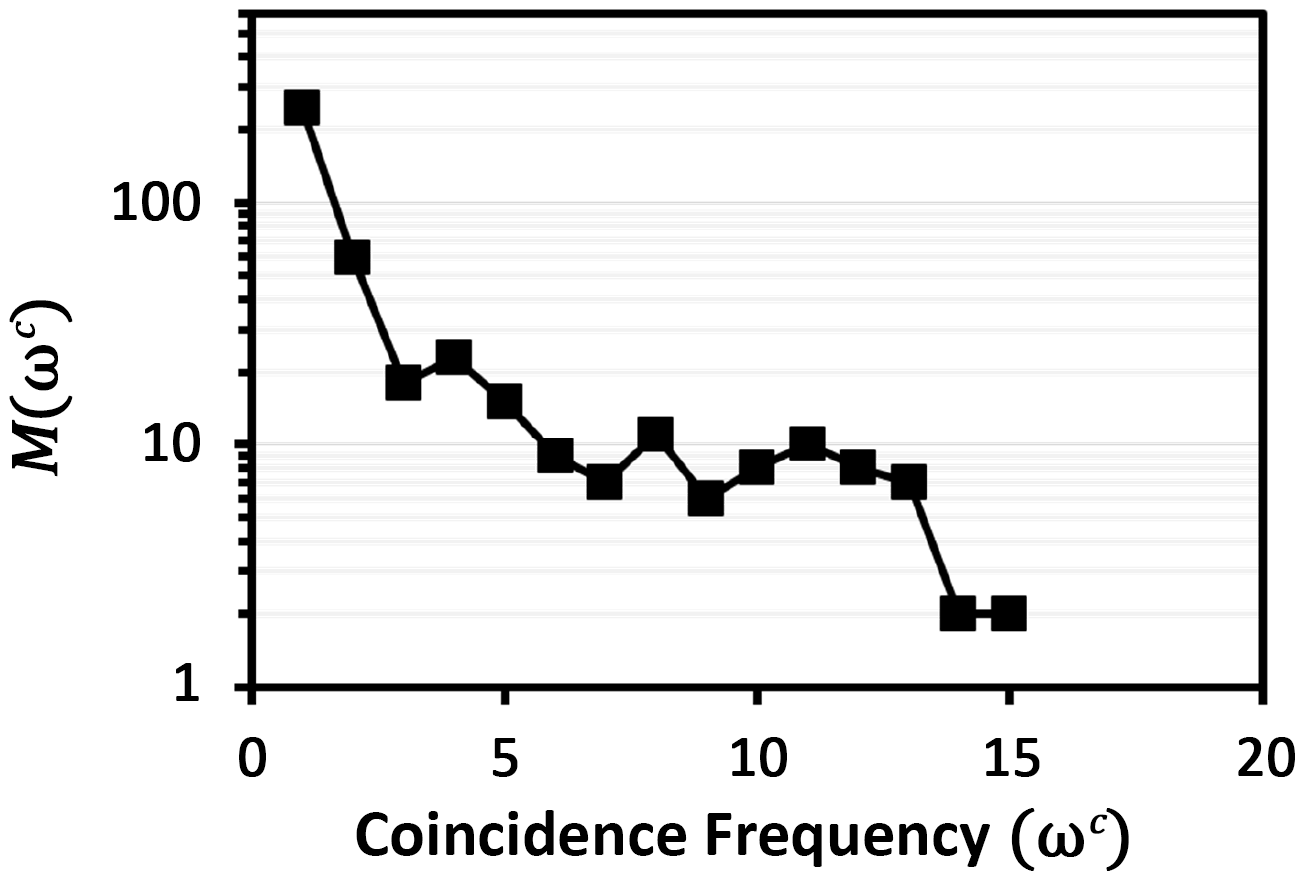

Supplement: Supplementary file 1 [file Image3.JPEG]

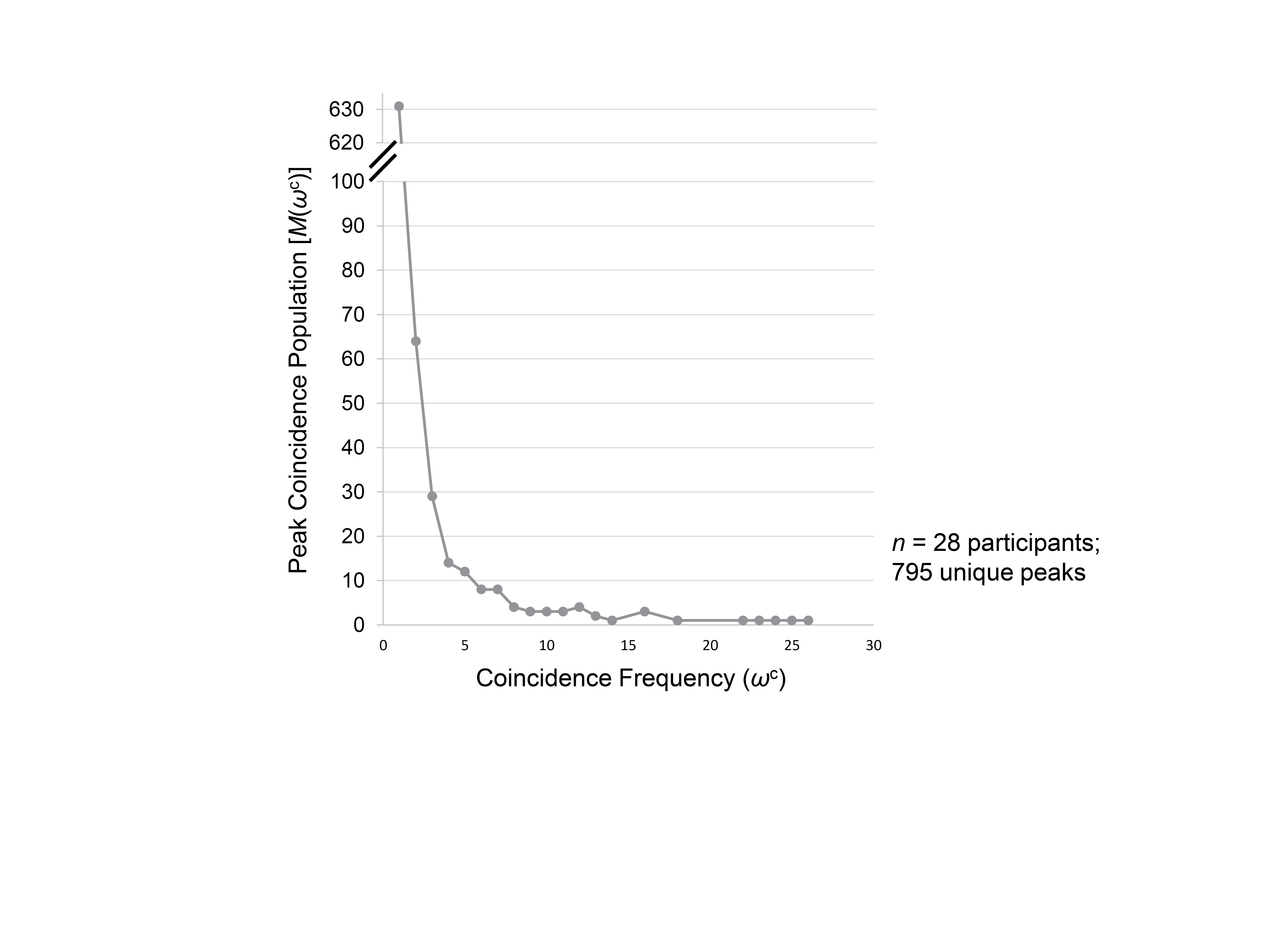

Supplement: Supplementary file 2 [file Image1.JPEG]

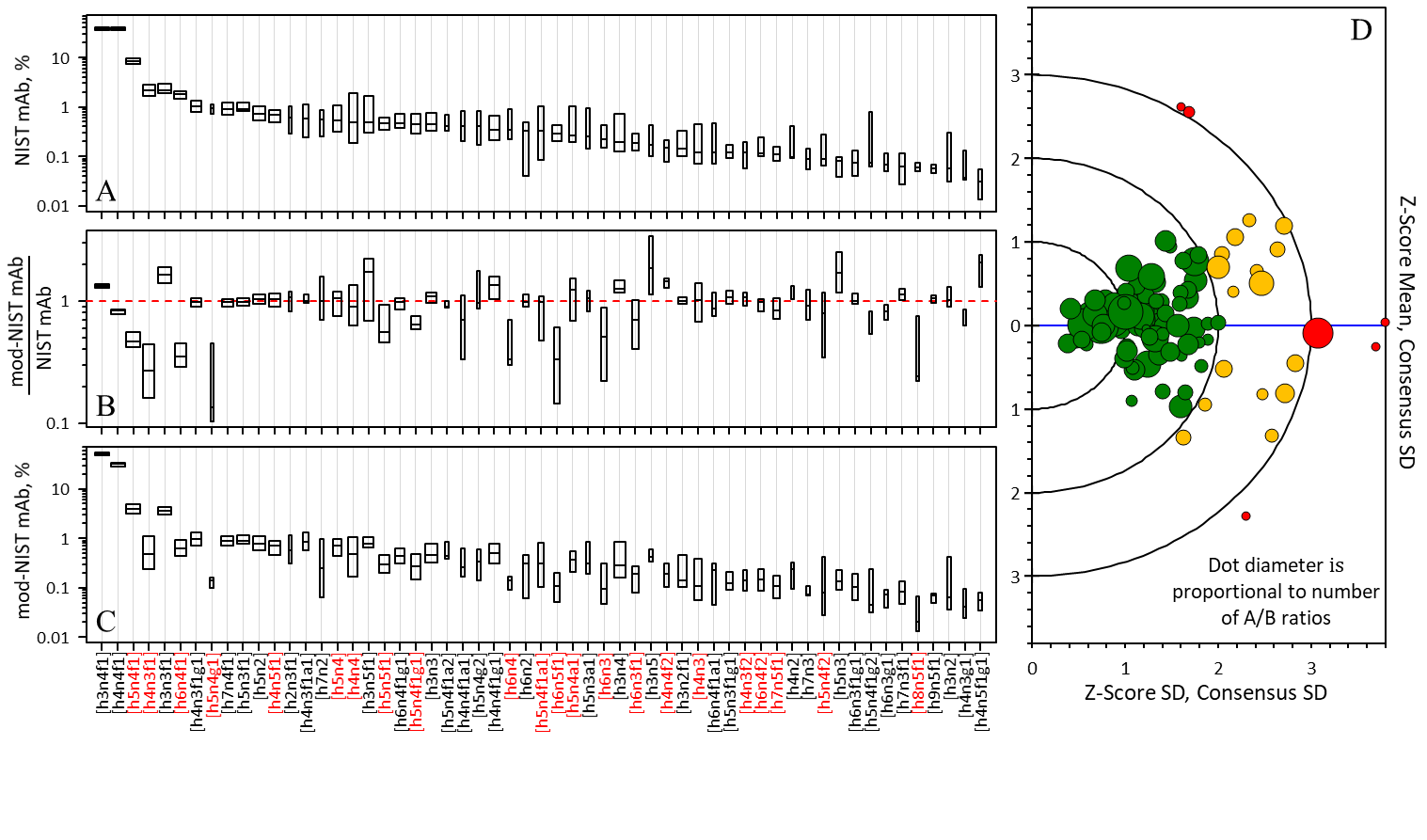

Supplement: Supplementary file 3 [file Image2.JPEG]
