# Supplementary material for: Interlaboratory Studies Using the NISTmAb to Advance Biopharmaceutical Structural Analytics
Source: Front Mol Biosci. 2022 May 5;9:876780. doi: 10.3389/fmolb.2022.876780 (PMC9117750; doi:10.3389/fmolb.2022.876780)
Supplement: Supplementary file 4 [file DataSheet1.docx]

Supplementary Material

## Supplementary Figures

**Supplementary Figure S1.** **Consensus of Peaks Reported in the pH Stress Sample.** For each unique peak reported as new, missing, or changed in the pH Stress Sample, the coincidence frequency (ω^c^) (number of participants reporting a given peak) and peak coincidence population values [*M*(ω^c^)] (number of unique peaks with the given coincidence frequency) were calculated.

This figure was adapted from Mouchahoir *et al*., 2021 (<https://pubs.acs.org/doi/10.1021/jasms.0c00415>), with permission from ACS Publications; further permissions related to this material should be directed to ACS.

**Supplementary Figure S2.** **Summary of results for the glycosylation interlaboratory study**.

Box plots for A) mod‑NISTmAb, B) mod‑NISTmAb/NISTmAb ratio, and C) NISTmAb PS 8670. Glycan compositions in the x-axis are sorted in order of decreasing NISTmAb abundances; red compositions have dominant terminal β1,4‑gal glycan structure. The dashed red line in panel B denotes the expected 1.0 ratio when mod-NISTmAb and NISTmAb have the same glycan result. Glycan % abundance in the y-axis is in log scale. The central 50% mean distribution is depicted on each box, with the consensus median represented in the horizontal middle line and the square root of the number of laboratories that identified that glycan represented in the width of the box. D) Target plot summary of mod‑NISTmAb/NISTmAb ratios relative to the consensus medians. Each dot represents one set of results with the number of reported ratios proportional to its diameter. The dots are colored based on distance from (0,0): green if within two comparability units, yellow if between two and three units, and red if greater than three units. The average bias estimated as the mean of the "Z-score" values of the ratios is shown in the “Z-score Mean” axis. The variability of individual bias estimates, estimated as the Z-score standard deviation, is shown in the “Z-Score SD” axis.

Nomenclature for [glycan composition]: Small letters were used to avoid confusion with elements (*e.g*., hydrogen, nitrogen, fluorine): h=hexose, n=N-acetylhexosamine, f=deoxyhexose (*e.g*., fucose), a=NeuAc, g=NeuGc. For sulfonated glycans, S=sulfur. Number after the letter denotes the number of residues. For example, [h6n4f1a2] has 6 hexoses, 4 N-acetylhexosamine, 1 fucose, 2 NeuAc.

**Supplementary Figure S3.** **Sequence coincidence population** $\boldsymbol{M}\left( \boldsymbol{\omega}^{\boldsymbol{c}} \right)$ **vs. Coincidence Frequency (**$\boldsymbol{\omega}^{\boldsymbol{c}}$) **for sequences reported by the 15 laboratory HDX-MS cohort.** Black squares plot the observed $M\left( \omega^{c} \right)$ found in the cohort data.

Reprinted with permission from Hudgens JW, Gallagher ES, Karageorgos I, Anderson KW, Filliben JJ, Huang RY, Chen G, Bou-Assaf GM, Espada A, Chalmers MJ, Harguindey E, Zhang HM, Walters BT, Zhang J, Venable J, Steckler C, Park I, Brock A, Lu X, Pandey R, Chandramohan A, Anand GS, Nirudodhi SN, Sperry JB, Rouse JC, Carroll JA, Rand KD, Leurs U, Weis DD, Al-Naqshabandi MA, Hageman TS, Deredge D, Wintrode PL, Papanastasiou M, Lambris JD, Li S, Urata S. Interlaboratory Comparison of Hydrogen-Deuterium Exchange Mass Spectrometry Measurements of the Fab Fragment of NISTmAb. Anal Chem. 2019 Jun 4;91(11):7336-7345. doi: 10.1021/acs.analchem.9b01100. Epub 2019 May 14. PMID: 31045344; PMCID: PMC6745711.Copyright {2019} American Chemical Society.
